# Isolation and partial characterization of a new moderate thermophilic *Albidovulum* sp. SLM16 with transaminase activity from Deception Island, Antarctica

**DOI:** 10.1186/s40659-018-0210-7

**Published:** 2019-02-04

**Authors:** Sebastián L. Márquez, Jenny M. Blamey

**Affiliations:** 1 Fundación Científica y Cultural Biociencia, José Domingo Cañas 2280, Santiago, Chile; 2 Facultad de Química y Biología, Universidad de Santiago de Chile, Avenida Libertador Bernardo O´Higgins 3363, Santiago, Chile

**Keywords:** Antarctica, *Albiduvulum*, *Moderate thermophile*, *Amine*-*transaminase*

## Abstract

**Background:**

A moderately thermophilic, slightly halophilic, aerobic, Gram-stain negative, bacterial strain, SLM16, was isolated from a mixed of seawater–sand-sediment sample collected from a coastal fumarole located in Whalers Bay, Deception Island, Antarctica. The aim was to screen for thermophilic microorganisms able to degrade primary amines and search for amine transaminase activity for potential industrial application.

**Results:**

Identification and partial characterization of the microorganism SLM16 were carried out by means of morphological, physiological and biochemical tests along with molecular methods. Cells of strain SLM16 were non-motile irregular rods of 1.5–2.5 μm long and 0.3–0.45 μm wide. Growth occurred in the presence of 0.5–5.5% NaCl within temperature range of 35–55 °C and pH range of 5.5–9.5, respectively. The DNA G+C composition, estimated from *ftsY* gene, was 66% mol. Phylogenetic analysis using de 16S rRNA gene sequence showed that strain SLM16 belongs to the marine bacterial genus *Albidovulum.*

**Conclusion:**

Strain SLM16 is a moderate thermophilic Gram negative microorganisms which belongs to the marine bacterial genus *Albidovulum* and is closely related to *Albidovulum inexpectatum* species based on phylogenetic analysis. Additionally, amine-transaminase activity towards the arylaliphatic amine α-methylbenzylamine was detected.

## Introduction

Deception Island (62°57′S, 60°38′W) in Antarctica is the caldera of a currently active volcano located in the South Shetland Islands archipelago. This island is one of the most singular environments in Antarctica due to the abundance of geothermal activity including hot soils, hot springs and fumaroles that evidentiate the volcanic activity of this site. The presence of these anomalies provide ideal conditions for the growth and thrive of thermophilic and hyperthermophilic microorganisms. However, not many thermophilic microorganisms from Antarctica have been described so far [1], mainly endospore-forming bacteria belonging to the genus Bacillus have been reported. The discovery and study of new microorganisms from this continent, particularly thermophiles, is important not only for the contribution to the knowledge of biodiversity but as a source of novel biocompounds with potential biotechnological applications like thermozymes. In this work we described a new member of the genus Albidovulum with amine transaminase activity. This genus was proposed as a new bacterial genus, phylogenetically very closely related to *Rhodovulum* genus, almost one and a half decade ago, being its first member named *Albidovulum inexpectatum* [2], due to its unexpected physiological characteristics that differentiates it from genus *Rhodovulum*: slightly thermophilic (the most thermophilic within α-3 subclass of the phylum *Proteobacteria*) and non-photosynthetic. Since then, only one additional species has been reported and characterized: *Albidovulum xiamenense* [3]. Both organisms are moderately thermophilic/halophilic, catalase and cytochrome oxidase positive, Gram-stain negative rod-shaped bacteria isolated from hot springs. The finding of this new member, *Albidovulum* sp. SLM16 is novel since is the first microorganism of this genus isolated from Antarctica that present amine-transaminase activity.

In this context, enzymes for the production of optically pure amines, like amine-transaminases a particular class of ω-transaminases, have been widely investigated over the last years [4–7]. Nonetheless, amine-transaminase reported to date are from non-thermophilic microorganisms with the exception of the amine-transaminase of *Thermomicrobium roseum* [8] and other ω-transaminases not specific for amines [9, 10]. Therefore, potential industrial applications and stability advantages of thermophilic amine-transaminases have not yet been well studied. Here we present the initial identification of the amine-transaminase activity in *Albidobulum* sp. SLM16.

## Materials and methods

### Sample site and isolation

Sample collection was performed during the 52th Antarctic Chilean Scientific Expedition (ECA 52) on January 2016 from Deception Island, Whalers Bay (62°59′S, 60°34′W). The sample—a mixture of sand and sediment—was collected directly from the heat output of a fumarole at moderate depth during low tide using sterile screw-cap tubes in aerobic conditions. In situ measurements of temperature and pH were performed. Samples were transported and stored at 4 °C to maintain integrity. For enrichment cultures, a proper amount of sample was inoculated on Zobell Marine Broth 2216 medium (HiMedia Laboratories) at pH 7.6 medium and incubated for 24 h at 50 °C using orbital agitation (120 rpm). For the screening of microorganisms potentially able to utilize (*R*,*S*)-α-methylbenzylamine (MBA) as a sole carbon source, a modified half-diluted Zobell Marine Broth 2216 medium supplemented with 20 mM HEPES and 10 mM α-methylbenzylamine as inductor for amine-transaminase activity was further used for selection of microorganisms. Serial dilutions and spread-plate techniques were used for isolation of colonies. Isolated strain was maintained by routinely sub-culturing in fresh growth medium and additionally as 20% glycerol suspension and stored at − 80 °C.

### Morphological, physiological and biochemical characterization

Phase-contrast microscopy (Nikon Eclipse 80i) and scanning electron microscopy (SEM) (Hitachi TM3000) on fresh cultures during exponential-phase were used to study cell morphology, spore formation and presence of flagella. Samples for SEM were fixed in 2.5 glutaraldehyde containing 0.1 M sodium cacodylate buffer (pH 7.4), filtered (Isopore™Millipore 0.2 μm) and further dehydrated with ethanol followed by critical-point drying, attached to a stub and finally coated with gold. Motility was examined using the hanging drop method and light microscopy (Olympus CX31). Biochemical tests were performed using API 20E and API 20NE according to the indications of manufacturer. Enzyme activities were examined using APIZYM (bioMerieux’s) kit. Catalase and oxidase activities were examined by observing bubble formation in H_2_O_2_ solution (3% v/v) and color change of oxidase reagent (*N,N,N*′*,N*′-tetramethyl-p-phenylenediamine 1%, BD BBL™) from uncolored to dark-purple, according to the method proposed by Kovacs [11], respectively. Gram-stain was determined by using Difco Gram-staining kit (BD Difco™ BBL™).

### DNA G+C content

The DNA G+C content was estimated by analyzing the universally conserved *ftsY* gene from the GTPase superfamily according to Fournier et al. [12].

### Optimal temperature and pH

Optimum temperature range for growth was determined measuring the optical density (OD_600_) of liquid cultures after 12 h incubation at temperatures ranging between 25 and 65 °C (in 5 °C increments) under aerobic conditions. The pH range for growth was examined in liquid cultures at 50 °C in a range of pH values from 5.5 to 9.5 using the following buffers: 20 mM 4-morpholineethanesulfonic acid for pH values 5.5–6.0, 20 mM 1,4-piperazinediethanesulfonic acid for pH values 6.5–7.0, 20 mM 4-(2-hydroxyethyl)piperazine-1-ethanesulfonic acid for pH values 7.5–8.0, 20 mM [(2-hydroxy-1,1-bis(hydroxymethyl)ethyl)amino]-1-propanesulfonic acid for pH values 8.5–9.0 and 3-(cyclohexylamino)-1-propanesulfonic acid for pH 9.5. Each buffer was adjusted to the desired pH value using HCl or NaOH.

### Effect of NaCl concentration

Effect of salinity was examined from 0.5 to 9% NaCl in half-diluted Zobell 2216 medium supplemented with the appropriate amount of NaCl. For 0% NaCl, medium was prepared from scratch omitting the sodium chloride. Unless otherwise specified, all the morphological and physiological analysis were performed on triplicate cultures grown in Zobell marine broth 2216 medium (HIMEDIA^®^).

### Antibiotic susceptibility

Antibiotic susceptibility was tested by using disc diffusion method on agar plates. The following sensidiscs (BBL™ Sensi-Disc™) were tested: ampicillin (10 μg), erythromycin (15 μg), tetracycline (30 μg), chloramphenicol (30 μg), amikacin (30 μg), nitrofurantoin (300 μg), ciprofloxacin (5 μg), sulfa-trimethoprim (25 μg), levofloxacin (5 μg), gentamicin (10 μg), cefazolin (30 μg), cefotaxime (30 μg), cefuroxime (30 μg), imipenem (10 μg), meropenem (10 μg), ceftriaxone (30 μg). Results were read after 12 h incubation at 37 °C. These measurements were carried out a single time.

### Phylogenetic analysis

Genomic DNA of strain SLM16 was obtained from an isolated colony by means of phenol–chloroform extraction as described by Rainey et al. [13], with minor modifications. Whole genome sequencing was performed on Illumina Miseq platform using Nextera XT DNA libraries at Georgia Genomics Facility (Georgia, USA) (data not shown). Trimmed Illumina reads were assembled using Velvet [14] assembler (version 1.2.1). Complete 16S rRNA gene sequence was predicted from the draft assembly using RNAmmer [15] software. Multiple sequence alignment for phylogenetic analysis was performed using MUSCLE [16] and further curated using Gblocks to eliminate poorly aligned regions [17]. Phylogenetic relationship among 16S rRNA gene sequences from *Albidovulum* close related genera was estimated using maximum likelihood statistical method implemented in MEGA (v.7) software for molecular evolutionary analysis [18]. Discrete Gamma distribution was used to model evolutionary rate differences among sites (5 categories, +G parameter = 0.2672). The rate variation model allowed for some sites to be evolutionarily invariable (+I, 49.4% sites). The reliability of the tree topology was assessed by using bootstrapping analysis based on 1000 replicates.

### Transaminase activity

Amine-transaminase activity was assayed on crude extract of strain SLM16, using the acetophenone assay reported by Schätzle et al. [19]. Assays were carried out at 50 °C for 4 min, using 2.5 mM (*S*)-α-methylbenzylamine and 2.5 mM pyruvate as substrate pairs in 1 ml potassium phosphate buffer 50 mM (pH 7) adding enzyme extract (100 μg) in the reaction mixture. Acetophenone formation was detected at 245 nm. One unit of activity was defined as the amount of enzyme that produces 1 μmol of acetophenone from (*S*)-α-methylbenzylamine in 1 min.

## Results and discussion

### Sample site and isolation of a moderate thermophilic bacteria

The microorganism designated SLM16 was isolated from a seawater-sediment sample collected from coastal fumaroles emerging at the shore at Whalers Bay in Deception Island, Antarctica. These fumaroles are only visible at low tides and are fully covered by the sea at high tides, which means that thermal gradients are generated all over the shore, including the sampling site. Temperature and pH values determined in situ at the time of sampling were 50 °C and pH 6.0–6.5, respectively. After enrichment of the sample in Zobell Marine Broth 2216 medium, a mixed culture formed by different types of rods was obtained. The mixed culture was further transferred to a selective media containing 5 mM α-methylbenzylamine as an alternative source of nitrogen. By using serial dilutions (up to 10^−20^) and spread plate techniques, it was possible to obtain easily differentiable colonies. Isolate SLM16 was obtained from a small, white, circular colony.

### Morphological, physiological and biochemical characterization

**Fig. 1:**
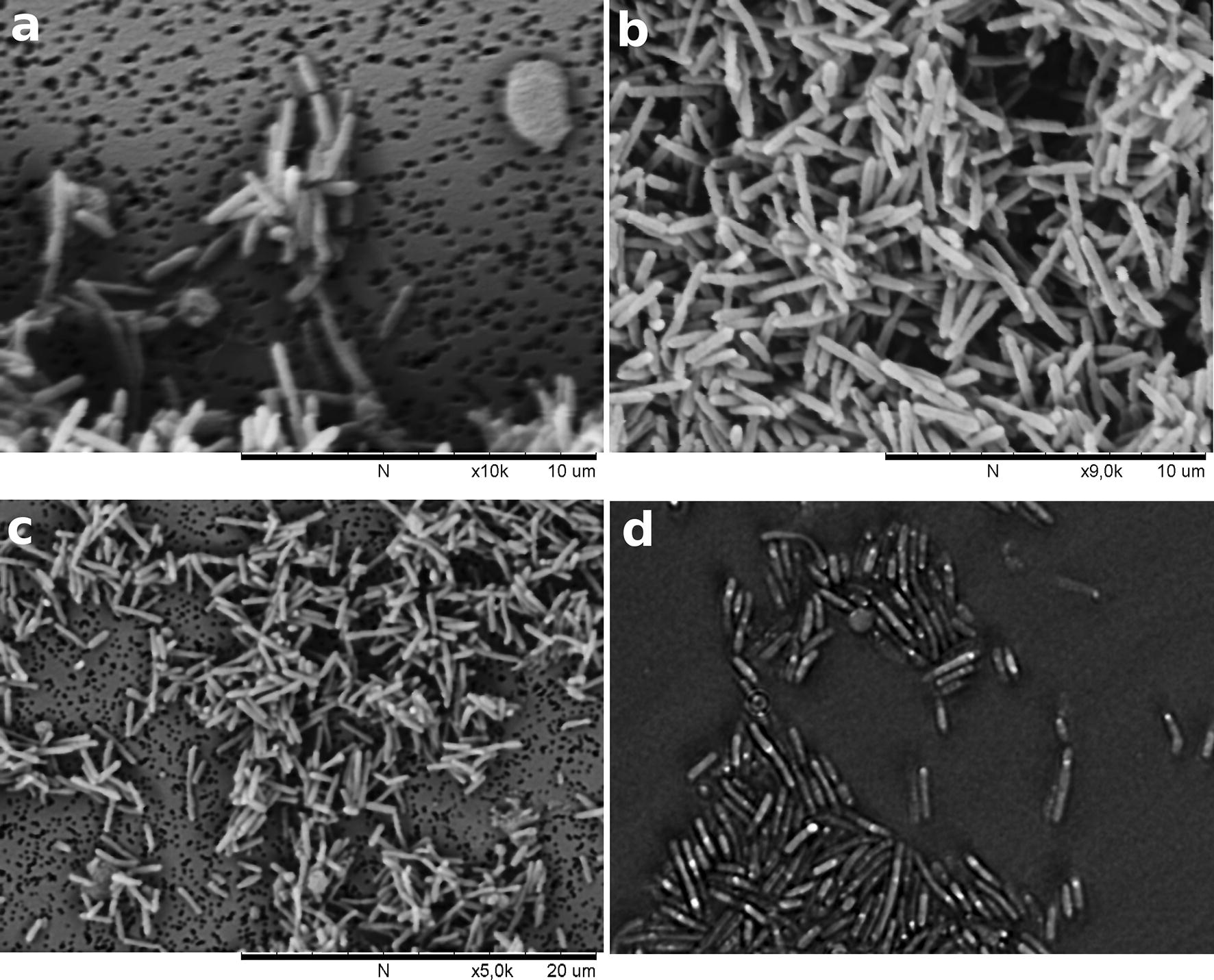
Scanning electron micrographs of strain SLM16, **a** ×10000 magnification **b** ×9000 magnification **c** ×5000 magnification. **d** Phase-contrast micrograph of strain SLM16. White points within the body of the microorganisms correspond to refractive inclusion bodies formed during starvation

After 12–24 h of incubation at 50 °C on solid 2216 medium, strain SLM16 produced circular, convex, entire margin, non-pigmented (white) colonies that were approximately 1–2 mm in diameter. Older colonies (24–48 h) developed a brownish color. Cell morphology was determined by means of phase-contrast microscopy and scanning electron microscopy after 12–24 h of incubation on liquid Zobell Marine Broth 2216 medium. Cells of strain 2216 were short rod-shaped, 1.5–2.5 μm in length and 0.3–0.45 μm in width (Fig. 1). Motility, presence of flagella and sporulation were not observed and this was further confirmed by the absence of sporulation, motility and chemotaxis related genes in the genome sequence which was obtained but it is not currently publicly available. Gram staining showed that the cells of SLM16 were negative stained. In starving conditions of strain SLM16, meaning when several days has passed since the initial inoculation of the media with the microorganism, it was possible to observe the formation of refractive inclusion bodies that may be confused with endospores (Fig. 1d). We could corroborate this because these inclusion bodies did not stain with endospore specific staining (malachite green solution).

**Fig. 2:**
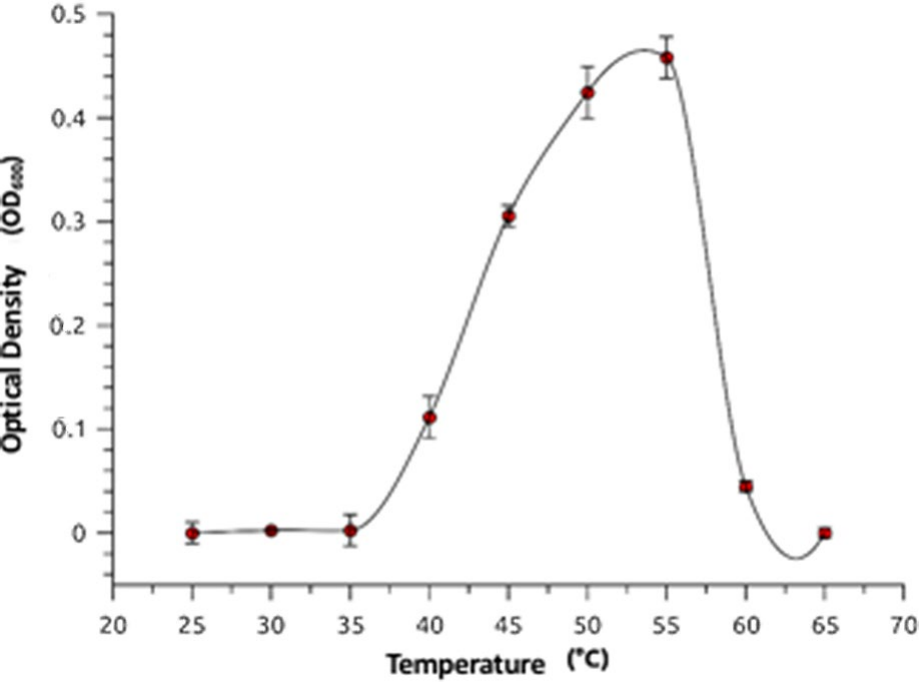
Optimum growth temperature of *Albidovulum* sp. SLM16. Temperature range for growth was determined measuring the optical density (OD_600_) of cultures after 12 h incubation at temperatures ranging between 25 and 65 °C. Error bars represent the standard deviation of three independent measurements

**Fig. 3:**
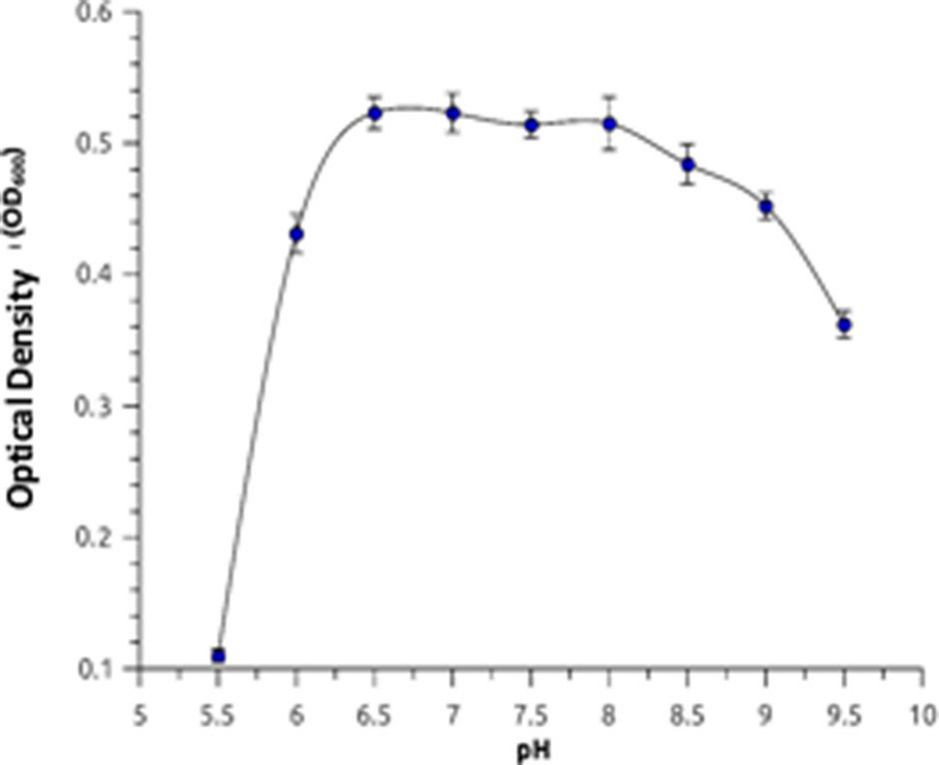
Optimum pH for growth of *Albidovulum* sp. SLM16. Culture media was incubated with different buffers: pH 5.5–6.0 (MES); pH 6.5–7.0 (PIPES); pH 7.5–8.0 (HEPES); pH 8.5–9.0 (TAPS); pH 9.5 (CAPS). As control for the measurements it was used culture medium without inoculation. Error bars represent the standard deviation of three independent measurements

The optimum temperature range for growth of SLM16 was around 50–55 °C. No significant growth was observed at 25–30 °C or 60–65 °C (Fig. 2). Growth was observed over a wide range of pH values from 5.5 to 9.5, exhibiting an optimal in the range of 6.5–8.0. Growth also occurred in presence of 0.5–5.5% NaCl being the optimal in the range 1–3% and no growth was observed at 0% NaCl or at concentrations higher than 5.5% NaCl, suggesting that strain SLM16 is slightly halophilic (Fig. 3).

Microorganism was catalase and cytochrome oxidase positive. According to API20 E and API20 NE results, strain SLM16 was positive for β-galactosidase (substrates: para-nitrophenyl-β-d-galactopyranoside, ortho-nitrophenyl-β-d-galactopyranoside), l-tryptophane deaminase, gelatinase, β-glucosidase (aesculin hydrolysis), reduction of nitrates to nitrites (NO_2_^−^) and acetoin production. Negative results were obtained for arginine dihydrolase, lysine decarboxylase, ornithine decarboxylase, urease, indol production (from tryptophan), glucose fermentation, citrate utilization, H_2_S production and reduction of nitrates to nitrogen (N_2_). All fermentation/oxidation tests for carbohydrates were negative in API20 E strips. Carbohydrate assimilation test according to API20 NE was positive for d-glucose, d-maltose, malic acid and weakly positive for d-mannitol and adipic acid. Negative assimilation results were obtained for l-arabinose, d-mannose, *N*-acetyl-glucosamine, potassium gluconate, capric acid, trisodium citrate and phenylacetic acid. According to API ZYM results, strain SLM16 was also positive for alkaline phosphatase, esterase (C4), esterase lipase (C8), leucine arylamidase, valine arylamidase, acid phosphatase, naphthol-AS-BI-phosphohydrolase, α-galactosidase, β-galactosidase (substrate: 2-naphthyl-β-d-galactopyranoside), α-glucosidase, β-glucosidase, weakly positive for cystine arylamidase and negative for esterase (C14), trypsin, α-chymotrypsin, β-glucuronidase, *N*-acetyl-β-glucosaminidase, α-mannosidase, α-fucosidase.

Antibiotics susceptibility tests showed that strain SLM16 is sensitive to ampicillin, erythromycin, tetracycline, chloramphenicol, ampicillin, nitrofurantoin, levofloxacin, gentamicin, amikacin, cefazolin, cefotaxime, cefuroxime, meropenem, ceftriaxone, intermediate to ciprofloxacin and resistant to sulfa-trimethoprim.

### Transaminase activity

**Fig. 4:**
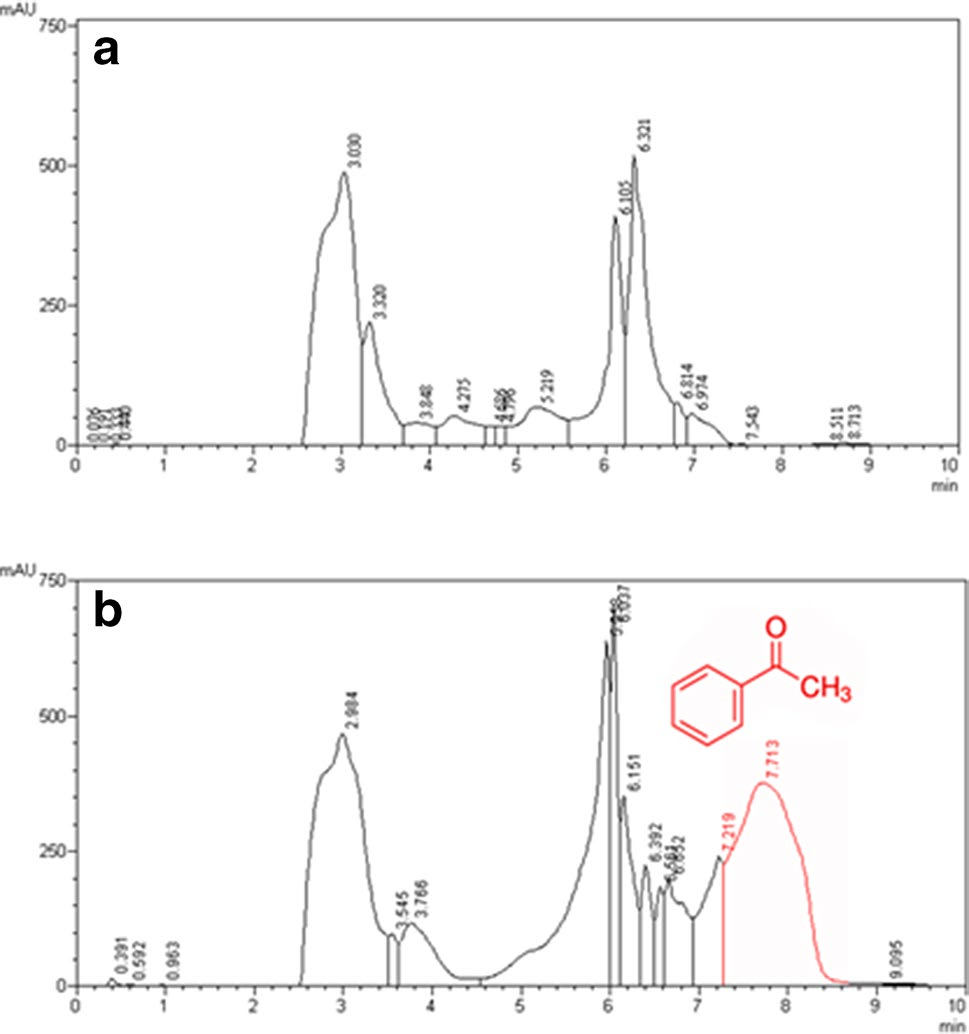
Detection of acetophenone by HPLC in crude extract of the strain SLM16 induced by (*S*)-α-MBA. **a** Negative control: products formed on the reaction when (*S*)-α-MBA is not present as sole nitrogen source. **b** Production of acetophenone in the presence of (*S*)-α-MBA and pyruvate. The images show the elution profiles of samples (λ = 245 nm)

Amine-transaminase activity assay was positive. A constant increase in the absorbance was observed after the addition of 100 μg crude extract from strain SLM16 to the reaction mixture containing 2.5 mM (*S*)-α-methylbenzylamine, indicating the transformation of this substrate into acetophenone. Specific activity of the potential amine-transaminase enzyme was 0.017 U/mg. The standard assay was defined as: solution mixture volume (1 ml) composed by Tris–HCl 100 mM (pH 8.0), (*R*) or (*S*)-α-MBA 1 mM, pyruvate 1 mM and PLP 10 μM incubated for 3 min at 50 °C. The reaction was initiated by adding 100 μg of crude extract to register the increase in absorbance for 1 min at 245 nm. As negative control the reaction mixture without substrate was used. Acetophenone production was confirmed by means of HPLC analysis (Fig. 4). This enzyme is currently being purified and characterized due to its biotechnological relevance.

### Phylogenetic analysis

**Fig. 5:**
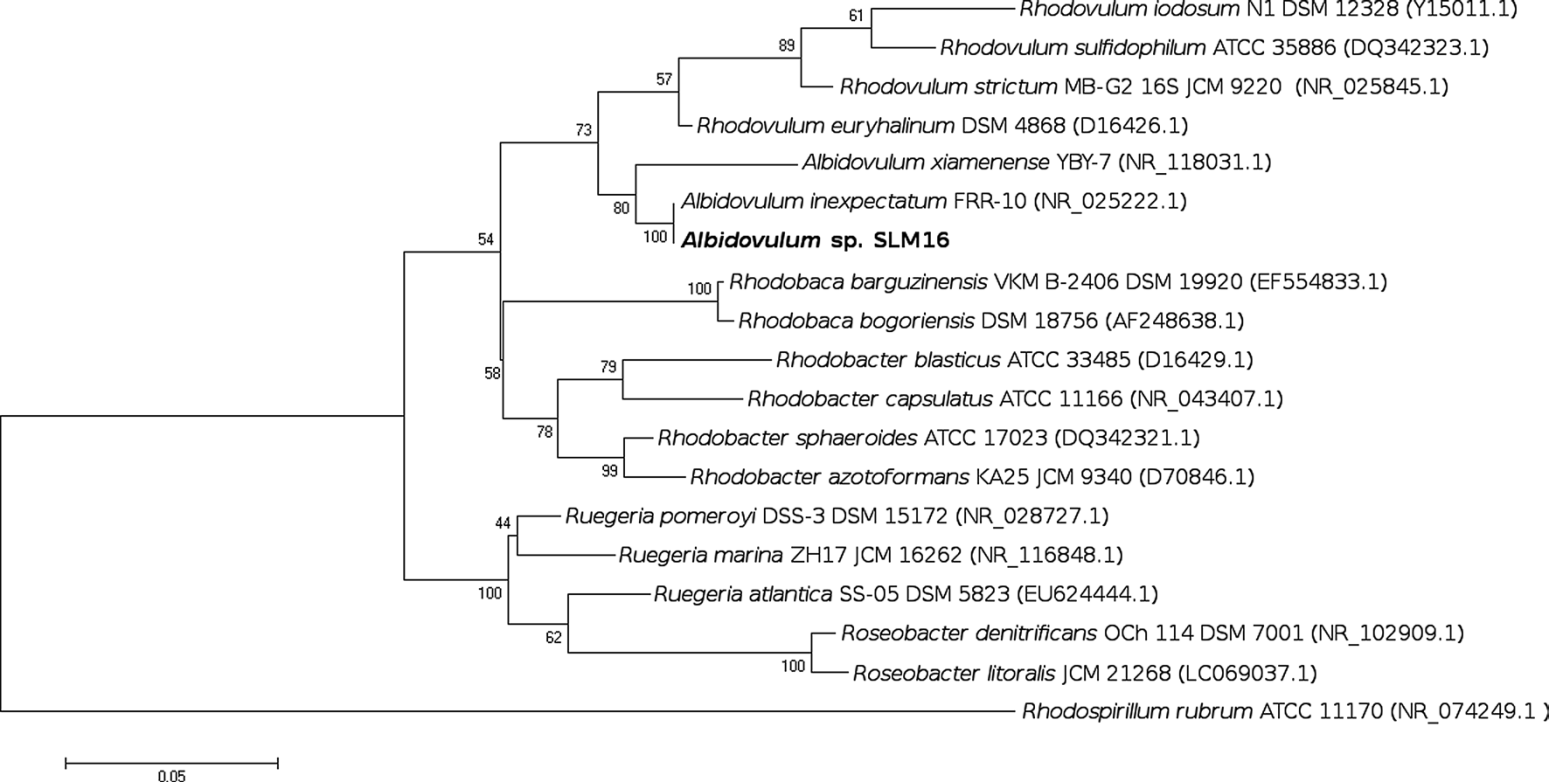
Maximum likelihood tree based on almost-complete 16S rRNA gene sequences showing the phylogenetic placement of strain SLM16 within the family *Rhodobacteraceae* build using MEGA7 software. *Rhodospirillum rubrum* was used as outgroup to root the tree. Genetic distances were estimated from Hasegawa-Kishino-Yano model. Percentages of bootstrap replicates (1000) supporting the topology of the tree are given above each branch. Genbank accession numbers are given in parenthesis

The complete 16S rRNA sequence (1457 bp long) from strain SLM16 was found in the genome assembly by means of RNAmmer. A multiple sequence alignment was then built between this sequence along with other complete and partial 16S rRNA gene sequences from representative species of *Albidovulum* close-related genera from the *Rhodobacteraceae* family. The phylogenetic reconstruction built using the Maximum Likelihood method revealed that 16S rRNA gene of strain SLM16 clusters together with *A. inexpectatum* and *A. xiamenense* (Fig. 5), showing a closer evolutionary distance to the former. This cluster is highly supported by bootstrap confidence level of 100%. The 16S rRNA gene sequences from strain SLM16 and *A. inexpectatum* were almost identical, sharing 99% identity over the length of the partial sequence of the latter (1409 bp). *Albidovulum* cluster falls within the radiation of *Rhodovulum* and *Rhodobaca* genera, being more closely related to the former, as expected according to the analysis reported Albuquerque et al. [2].

The results of the phylogenetic and physiological analysis reported so far, allowed us to assign strain SLM16 to the genus *Albidovulum* and designated it *Albidovulum* sp. SLM16. High similarity between the 16S rRNA gene sequence of *Albidovulum* sp. SLM16 and *A. inexpectatum* (99%) along with very similar values in terms of morphological and physiological characteristics like cell dimensions, optimum temperature, pH and NaCl concentration suggest that the former could belong to the same species. However, biochemical characteristics (Table 1) like differences in carbohydrates assimilation, the presence of enzymes like gelatinase that has not been reported to be present in *A. inexpectatum* among others enzymes that were reported as weakly positive for the latter that resulted positive in strain SLM16 suggest metabolic differences. As whole genome sequence of *A. inexpectatum* is not freely available genome-wide comparison as Average Nucleotide Identity (gANI) could not be made.

**Table 1:** Morphological, biochemical and physiological comparison between strain SLM16 and the two species of *Albidovulum* reported to date

Characteristics	Strain SLM16	*A. inexpectatum* FRR-10	*A. xiamenense* YBY-7
Cell length/width (μm)	1.5–2.5/0.3–0.45	1.4–2.2/0.4–0.6	2–6/0.4–0.6
GC content (%mol)	66.0^a^	63.6	70.6
Temperature range (°C)	50–55	50	50–58
pH range	6.5–8.0	6.5–8.0	7.5–8.5
NaCl range (%w/v)	1–3	1–3	3
Motility	−	−	+
*Enzymes*			
Arginine dihydrolase	−	+	+
Lysine decarboxylase	−	+	+
Ornithine decarboxylase	−	+	+
Urease	−	+	+
Gelatinase	+	−	−
Esterase (C4)	+	±	±
Cystine arylamidase	±	+	±
Trypsin	−	±	±
α-Chymotrypsin	−	±	±
Naphthol-AS-BI-phosphohydrolase	+	ND	−
β-Glucuronidase	−	±	±
*Carbohydrates utilization*			
l-Arabinose	−	+	−
d-Mannose	−	+	+
Trisodium citrate	−	+	−

Data from *A. inexpectatum* FRR-10 and *A. xiamenense* YBY-7 were obtained from [1] and [2]

Temperature, pH and NaCl values correspond to the optimum values reported. (+, positive; −, negative; ±, weakly positive; ND, not determined)

^a^Estimated by analysis of *ftsY* gene

Additional experiments employing both strains under the same experimental conditions and genome comparisons are necessary for further differentiation at species level.

### Description of *Albidovulum* sp. SLM16

*Albidolvulum* sp. SLM16 forms short rod-shaped cells 1.5–2.5 μm long and 0.3–0.45 μm wide. Microorganism is catalase and oxidase positive, non-motile, non-flagellated, non-spore-forming and stains Gram-negative. Colonies on Zobell Marine Broth 2216 medium in agar are non-pigmented circular, entire margin and convex. Older colonies develop a brownish coloration. Moderately thermophilic, the optimum temperature range for growth is 50–55 °C and the optimum pH range is 6.5–8.0. Slightly halophilic, NaCl concentration between 1 and 3% was optimal for growth. DNA G+C content estimated by *ftsY* gene analysis was 66 %mol. Positive for β-galactosidase, l-tryptophane deaminase, gelatinase, β-glucosidase, reduction of nitrates to nitrites and acetoin production. Also positive for alkaline phosphatase, esterase (C4), esterase lipase (C8), leucine arylamidase, valine arylamidase, acid phosphatase, naphthol-AS-BI-phosphohydrolase, α-galactosidase, β-galactosidase (substrate: 2-naphthyl-β-d-galactopyranoside), α-glucosidase, β-glucosidase and weakly positive for cystine arylamidase according to APIZYM test. The microorganism is resistant to the antibiotic sulfa-trimethoprim. Additionally, *Albidovulum* sp. SLM16 possesses amine-transaminase activity demonstrated by the specific enzymatic activity measurements and corroborated by HPLC analysis.

## Conclusion

A new moderately thermophilic microorganism, *Albidovulum* sp. SLM16, was isolated from a fumarole located in Whalers Bay, Deception Island, Antarctica, and characterized by means of traditional methods. This study contributes to the knowledge of bacterial diversity in geothermal sites of Antarctica. *Albidovulum* sp. SLM16 is the first microorganism of this genus isolated from the Antarctic continent. To the best of our knowledge, this is the first Antarctic microorganism possessing amine-transaminase activity reported to date and, also, one of the few thermophilic microorganisms with this biotechnological relevant characteristic.

## Abbreviations

MBA: methyl benzyl amine
SEM: scanning electron microscope
HEPES: 4-(2-hydroxyethyl)-1-piperazineethanesulfonic acid
gANI: genomic average nucleotide identity
HPLC: high performance liquid chromatography
*ftsY*: signal recognition particle receptor
NaCl: sodium chloride

## References

[1] Satyanarayana T, Kawarabayasi Y, Littlechild J (2013). Thermophilic microbes in environmental and industrial biotechnology: biotechnology of thermophiles. Thermophilic Microbes Environ..

[2] Albuquerque L, Santos J, Travassos P, Nobre MF, Rainey FA, Wait R (2002). *Albidovulum inexpectatum* gen. nov., sp. nov., a nonphotosynthetic and slightly thermophilic bacterium from a marine hot spring that is very closely related to members of the photosynthetic genus *Rhodovulum*. Appl Environ Microbiol..

[3] Yin D, Xiao J, Ao J, Ai C, Chen X (2012). *Albidovulum xiamenense* sp. nov., a moderately thermophilic bacterium from a terrestrial hot spring. Int J Syst Evol Microbiol..

[4] Turner NJ, Truppo MD (2013). Synthesis of chiral amines using transaminases. Sustain Catal..

[5] Mathew S, Yun H (2012). ω-Transaminases for the production of optically pure amines and unnatural amino acids. ACS Catal..

[6] Höhne M, Bornscheuer UT (2012). Application of Transaminases. Enzym Catal Org Synth..

[7] Steffen-Munsberg F, Vickers C, Thontowi A, Schätzle S, Meinhardt T, SvedendahlHumble M (2013). Revealing the structural basis of promiscuous amine transaminase activity. ChemCatChem..

[8] Mathew S, Deepankumar K, Shin G, Hong EY, Kim B-G, Chung T (2016). Identification of novel thermostable ω-transaminase and its application for enzymatic synthesis of chiral amines at high temperature. RSC Adv..

[9] Mathew S, Nadarajan SP, Chung T, Park HH, Yun H (2016). Biochemical characterization of thermostable ω-transaminase from *Sphaerobacter thermophilus* and its application for producing aromatic β- and γ-amino acids. Enzyme Microb Technol.

[10] Chen Y, Yi D, Jiang S, Wei D (2016). Identification of novel thermostable taurine–pyruvate transaminase from *Geobacillus thermodenitrificans* for chiral amine synthesis. Appl Microbiol Biotechnol.

[11] Kovacs N (1956). Identification of *Pseudomonas pyocyanea* by the oxidase reaction. Nature..

[12] Fournier PE, Suhre K, Fournous G, Raoult D (2006). Estimation of prokaryote genomic DNA G+C content by sequencing universally conserved genes. Int J Syst Evol Microbiol.

[13] Rainey F, Ward-Rainey N, Kroppenstedt RM, Stackebrandt E (1996). The genus Nocardiopsis represents a phylogenetically coherent taxon and a distinct actinomycete lineage: proposal of Nocardiopsaceae fam. nov. Int J Syst Bacteriol..

[14] Zerbino DR, Birney E (2008). Velvet: algorithms for de novo short read assembly using de Bruijn graphs. Genome Res.

[15] Lagesen K, Hallin P, Rødland EA, Stærfeldt HH, Rognes T, Ussery DW (2007). RNAmmer: consistent and rapid annotation of ribosomal RNA genes. Nucleic Acids Res.

[16] Edgar RC (2004). MUSCLE: multiple sequence alignment with high accuracy and high throughput. Nucleic Acids Res.

[17] Talavera G, Castresana J (2007). Improvement of phylogenies after removing divergent and ambiguously aligned blocks from protein sequence alignments. Syst Biol.

[18] Kumar S, Stecher G, Tamura K (2016). MEGA7: molecular evolutionary genetics analysis version 7.0 for bigger datasets. Mol Biol Evol.

[19] Schätzle S, Höhne M, Redestad E, Robins K, Bornscheuer UT (2009). Rapid and sensitive kinetic assay for characterization of ω-transaminases. Anal Chem.

